# Relationship between Selected Micronutrient Concentrations, Total Antioxidant Status, Pain Severity, and the Image of ^1^H MR Spectroscopy in Degenerative Spine Disease: A Case-Control Study

**DOI:** 10.3390/jcm11195586

**Published:** 2022-09-23

**Authors:** Marta Jakoniuk, Magdalena Biegaj, Jan Kochanowicz, Tomasz Łysoń, Agnieszka Lankau, Marianna Wilkiel, Katarzyna Socha

**Affiliations:** 1 Department of Invasive Neurology, Medical University of Białystok, M. Skłodowskiej-Curie 24a Street, 15-276 Białystok, Poland; 2 Department of Bromatology, Medical University of Białystok, Mickiewicza 2D Street, 15-222 Białystok, Poland; 3 Department of Neurology, Medical University of Białystok, M. Skłodowskiej-Curie 24a Street, 15-276 Białystok, Poland; 4 Department of Integrated Medical Care, Medical University of Białystok, M. Skłodowskiej-Curie 7a Street, 15-096 Białystok, Poland

**Keywords:** antioxidant status, zinc, copper, selenium, manganese, degenerative spine disease

## Abstract

Osteoarthritis of the spine is a common disease and constitutes a significant problem in highly developed countries. Due to the aging of the population, the number of patients with advanced degenerative changes continues to grow. Trace elements and antioxidant status may play an active role in the pathogenesis of osteoarthritis of the spine. The aim of this study was to assess the relationship between total antioxidant status (TAS), the concentration of selected elements (Zn, Cu, Se, Mn) in the serum, nutritional and environmental factors, the severity of pain, and images of degenerative changes in the spine demonstrated in proton magnetic resonance spectroscopy (^1^H MRS). The study included 90 patients with degenerative spine disease and 40 healthy people. Serum Zn, Cu, Se, and Mn concentrations were determined by atomic absorption spectrometry. The Cu/Zn molar ratio was calculated. TAS was determined spectrophotometrically using a ready-made Randox kit. The severity of spine pain was assessed using the visual analogue scale VAS. In the ^1^H MRS study, the fat/water ratio was determined in L1 and L5 vertebral bodies and in the L4/5 intervertebral disc. In patients with osteoarthritis of the spine, there was a reduced concentration of Zn and Cu in the serum (0.77 ± 0.22 mg/L, 1.1 ± 0.35 mg/L) compared to the control group (0.83 ± 0.13 mg/L, 1.25 ± 0.41 mg/L, respectively). In the study group, we also observed a significantly lower Cu concentration in smokers (1.07 ± 0.35 mg/L) compared to non-smokers (1.17 ± 0.34 mg/L). A relationship between the female sex and a decreased concentration of TAS in the serum, both in people with degenerative spine disease and in healthy people, has been demonstrated. In patients with serum TAS levels below the reference values, the fat/water ratio was higher in the L5 spine shaft, while in those with elevated Mn levels, the value was higher in the L4/L5 intervertebral disc, which indicates a greater degree of degeneration in both cases. Patients with normal serum Cu concentration experienced lower pain intensity assessed in the VAS scale. The study showed some abnormalities in serum trace element levels and TAS in patients with osteoarthritis of the spine.

## 1. Introduction

Osteoarthritis of the spine is a chronic and progressive disease that significantly reduces the quality of life of patients. The disease is characterized by premature wear and degeneration of the tissues that make up the functional joints of the vertebrae. It is the most common cause of back pain, and each year, 266 million people worldwide (3.63%) suffer from osteoarthritis of the lumbar spine and low back pain. The highest incidence is found in Europe (5.7%), while the lowest estimated incidence is in Africa (2.4%). Worldwide, 403 million (5.5%) people annually suffer from symptomatic disc degeneration, and 103 million (1.41%) people suffer from spinal stenosis. Low- and middle-income countries have four times more cases than high-income countries [1]. It is estimated that in Poland, about two million people suffer from osteoarthritis of the spine, and it is the most common reason for declaring disability. Severe pain associated with the disease is often the reason for absenteeism from work, which is a socio-economic problem. Clinical studies have shown that the disease affects 25–30% of people aged 45–64, 60% of people over 60 years of age and 80% of people over 80 years of age [2]. The disease can also begin in adults under 40 years of age. More and more studies indicate the presence of degenerative changes in the spine in people under 30 years of age, which is confirmed by magnetic resonance imaging (MRI). In a study by Cheung et al. conducted on 1043 volunteers between 18 to 55 years of age, 40% of people under the age of 30 had lumbar disc degeneration. The incidence of lesions increased gradually to over 90% between the ages of 50 and 55. The most commonly affected levels are L4–L5 and L5–S1 [3,4].

Currently, there are no effective methods of causal treatment: only symptomatic treatment is used, consisting of painkillers, anti-inflammatory drugs, muscle relaxants, B vitamins, and rehabilitation. Sometimes, the course of the disease is so severe that it requires the use of steroids and antidepressants. These treatments are often insufficient, and as the disease progresses, the patients require surgery [5]. The main reasons for the development of degenerative spine disease are advanced age, obesity, occupation, and past injuries. Taking into account the fact that, as a result of technological improvements and progressive computerization, the level of physical activity has decreased, it can be assumed that the frequency of degenerative changes in the spine will increase, especially among young people. Thus, the lumbar spine is particularly susceptible to degenerative changes. Under the influence of the above-mentioned risk factors, numerous changes in the spine may occur, leading to disruption of its functions and the development of degenerative spine disease. The degenerative process leads to the degradation of collagen and the breakdown of proteoglycans, reducing the water content within the tissues. As a consequence of this process, the intervertebral disc and cartilage of the intervertebral joints are damaged, which causes deterioration of the biomechanical properties of the spine and promotes microtraumatization of joint capsules and the ligament system [6]. Arthritis can affect any particular surface, including spine joints. The spinal consists of spinal motion segments—a complex of two intervertebral joints and one intervertebral disc—which degenerate over time and are a potential source of back pain. Degeneration forms vertebral osteophytes, facet joint osteoarthritis, and disc space narrowing. In particular, osteoarthritis of the spine indicates the presence of disc degeneration and the formation of osteophytes [5,7,8]. The developing inflammation, which is caused by, among other factors, oxidative stress, plays an important role in the above destructive processes of articular cartilage [9]. Smoking is an additional factor for generating oxidative stress. Toxic substances present in tobacco smoke increase the amount of free oxygen radicals [10].

Therefore, antioxidants play an important part in the diet of people suffering from degenerative spine disease; they are also responsible for an anti-inflammatory effect [11]. Antioxidant trace elements such as zinc (Zn), copper (Cu), selenium (Se), and manganese (Mn) are necessary for the proper functioning of the body. The homeostasis of these elements enables the proper activity of antioxidant enzymes, and thus offer an effective defense against oxidative stress. Zn and Cu, as components of Cu/Zn superoxide dismutase, have an antioxidant and anti-inflammatory activity and play a role in the regulation of the pathogenesis of inflammation-related diseases [12,13]. Zn supplementation has been shown to have a beneficial effect on bone tissue formation by affecting bone morphogenetic protein (BMP) [14]. Cu, as a component of lysyl oxidase—an enzyme responsible for the formation of cross-links in collagen and elastin—increases bone strength and helps maintain optimal bone health [15]. The key role of Se in human metabolism is associated with its presence in the glutathione peroxidases (GPx). Due to its antioxidant effect, it can also be of great importance in osteoarthritis of the spine, where free radicals affect the degeneration of articular cartilage and are the cause of the ongoing inflammation that accompanies this disease [16]. Mn plays an important role in bone formation, and currently, more attention is being paid to its importance in osteoarthritis of the spine [17]. 

In comparison, a new method for assessing the severity of degenerative changes in the spine is magnetic resonance spectroscopy (MRS), which, by assessing the fat-to-water ratio of a selected spine structure, can indirectly provide information about bone condition [18], while the VAS scale is the most frequently used tool for the subjective assessment of pain severity by the patient [19]. Therefore, the assessment of antioxidant status and the level of antioxidant minerals in relation to the results of MRS in patients with osteoarthritis of the spine may be crucial in the timely diagnosis and prevention of the development of this disease. It can be hypothesized that incorrect values of the antioxidants tested may correlate with a greater degree of degeneration of the spine. If antioxidant deficiencies are found, appropriate dietary modification or supplementation can be introduced, which will contribute to the improvement of the patients’ health.

The aim of the study was to assess the concentration of Zn, Cu, Se, Mn, and the total antioxidant status (TAS) in the serum in relation to the degenerative changes obtained in MRS in people with osteoarthritis of the spine.

## 2. Materials and Methods

### 2.1. Patients and the Control Group Characteristic

The study included 130 adults: 90 people aged 21 to 73 (mean: 48.17 ± 12.70 years) diagnosed by a neurologist and treated for degenerative spine disease at the “KENDRON” Neurological Outpatient Clinic in Białystok (Poland). Patients were qualified for the study by an experienced neurologist on the basis of symptoms, medical history, and neurological examination. The most common symptom reported by patients was pain in the lower back that worsened when sitting or standing and decreased when lying down. Other symptoms reported by the subjects were tenderness and stiffness of the spine joints, limited range of motion in the lumbar spine, weakness or numbness in the legs, and tingling in the legs. The pain and stiffness increased in the morning due to hours of inactivity. Among the examined patients, 67 people lived in a city of over 300,000 inhabitants, while the rest lived in surrounding small towns or in the countryside. Sedentary work was performed by 49 patients, mainly as drivers, dressmakers, and accountants. On the other hand, 41 people worked physically, most often as farmers, builders, or mechanics. The average BMI of patients was 28.1 ± 4.1. 

Patients were also qualified on the basis of their previously provided imaging examinations of the lumbar spine (X-ray, CT scan, MRI) that showed the presence of osteoarthritic degenerative alterations of discs, vertebral bodies, and intervertebral facets at the level of the lumbar spine. This allowed for an initial diagnosis of osteoarthritis of the spine and for the study exclusion of patients with changes such as disc herniation, spondyloarthropathy, infection, vertebral fracture, and abnormal growths such as cysts and tumors, neoplasm, myelopathy, and aortic aneurysm or dissection. The basis for inclusion in the study was the diagnosis of osteoarthritis of the lumbar spine. The exclusion criteria from the study also included other chronic diseases such as cancer, diabetes, cardiovascular diseases, endocrine diseases, and autoimmune diseases. 

The control group consisted of 40 healthy people who did not report any pain typical of degenerative spine disease nor other symptoms related to low back pain. In addition, all participants from the control group did not have a medical history of osteoarthritis of the spine or other osteoarthritis. The control group was appropriately selected in terms of sex, age, and body weight (the average BMI: 26.2 ± 3.8), as well as the percentage of smokers and non-smokers, compared to the group of people with degenerative spine disease. 

Patients and control group characteristics are presented in Table 1. Venous blood was collected from the subjects in vacuum test tubes (Becton Dickinson, France), and information on age, gender, smoking, and medications and/or dietary supplements were acquired.

### 2.2. Study Design

The severity of degenerative spine disease in patients was assessed on the basis of proton magnetic resonance spectroscopy (^1^H MRS). Examinations were performed with a SIEMENS Magneton Spectra apparatus (Germany) with a magnetic field strength of 3T at the Kendron NZOZ Outpatient Clinic. The patients underwent magnetic resonance spectroscopy (^1^H MRS) of the L1, L5 vertebral body and the L4/5 intervertebral disc. A standard protocol was used with two sequences: T2_TSE in the transverse plane and the sequences T2_TSE, T2_TIRM, and T1_TSE in the sagittal plane. The ^1^H-MRS planning was obtained on T2_SPC sequences (TR; 1300 ms; TE; 208 ms, section thickness 0.9 mm; intersection distance 0.2 mm; 56 sagittal sections; FOV (340 × 170); matrix size 288 × 192). The frontal and axial planes were reconstructed with the same technical parameters from these sequences; ^1^H-MRS was performed with a repetition time (TR) = 2000 ms and echo time (TE) = 50 ms using the single-voxel spectroscopy (SVS) previously strictly defined on the basis of a conventional MRI scan. Voxels (VOI, volume of interest) from the vertebral body L1 and L5 were analyzed, in which the lipid content and the hydration level of the intervertebral disc at L4/5 levels were assessed. A 12 × 12 × 12 mm voxel was used to obtain the spectrum from the vertebral body L1 and L5. The spectroscopic spectrum of the L4/L5 intervertebral disc was obtained using a 20 × 5 × 20 mm promissory note. The spectroscopic spectrum was analyzed using the Siemens dedicated SPECTROSCOPY application. Metabolites were obtained in the form of water peaks and lipids. The level of vertebral body L1, L5, and L4/L5 intervertebral disc steatosis was determined from the lipid-to-water ratio, where the ratio level was set at 1 for lipids. The fat-to-water ratio in the vertebral bodies L1, L5, and the intervertebral disc at L4/L5 was calculated. A higher value of the fat/water ratio indicated a higher degree of degeneration. Patients also assessed the severity of spine pain using the Visual Analog Scale (VAS). The VAS scale is usually a horizontal line with special markings. It has ten degrees, from 0, which means no pain at all, to 10, which means unbearable pain. The patient was asked to indicate the perceived pain intensity.

### 2.3. Sample Preparation and Biochemical Analyses

The blood samples were centrifuged for 10 min at approximately 1000× *g*. Serum was stored frozen at −20 °C. The serum samples were deproteinated using 1 mol/L spectral-grade nitric acid (Merck, Darmstadt, Germany) and 1% Triton X-100 (Sigma, Taufkirchen, Germany) was added, then mixed using vortex, and centrifuged for 10 min. The concentration of Zn was then determined in the supernatant; for Cu samples, it was determined after dilution in 0.1 mol/L nitric acid. The concentrations of Se and Mn were determined directly after dilution 1:1 with 0.2% Triton X-100.

The concentrations of mineral components were determined by the atomic absorption spectrometry method (AAS) with Zeeman background correction (Hitachi, Japan), using atomization in an acetylene-air flame (Zn) and the flameless technique with electrothermal atomization in a graphite tube (Cu, Se, and Mn). The detection limit was 0.01 mg/L for Zn, 0.54 µg/L for Cu, 1.66 µg/L for Se, and 0.11 µg/L for Mn.

Certified reference material (CRM) of human serum (Seronorm Trace Elements, Serum Level 1, 1801802, Sero AS, Hvalstad, Norway) was used to test the accuracy and precision of the analytical techniques. All the values of control samples were in agreement with the reference values. The precision of the methods for the determination of Zn, Cu, Se, and Mn was 3.2%, 2.4%, 2.6%, and 1.9%, respectively. The recovery for CRM was 101.3%, 99.2%, 98.6%, and 99.6%, respectively.

Total antioxidant status (TAS) was determined by spectrophotometric method using a kit of Randox reagents (Randox Laboratories Ltd., Crumlin, UK). Randox TAS Control (Bovine based serum) was used to test the accuracy of the method. 

Written informed consent was obtained from all study participants prior to the collection of blood samples. The study protocol was approved by the Ethics Committee of the Medical University of Bialystok (nr R-I-002/97/2017)

### 2.4. Statistical Analysis

Statistical analysis was performed using the Statistica v. 13 software. Data were tested for normal distribution by the Kolmogorov–Smirnov and the Lilliefors tests to assess whether there was a normal distribution of parameters. When the data distribution was normal, parametric tests were used. If the distribution showed asymmetry, non-parametric tests were used in further analyses. Differences between groups were evaluated by Student’s t-test for data with normal distribution and Mann–Whitney U-test for other data. Correlations were assessed with the Pearson or Spearman test, depending on the data distribution. Differences at the significance level *p* < 0.05 were considered statistically significant. The Kruskal–Wallis ANOVA test and post-hoc test were used to show differences in the degree of spinal degeneration and in the VAS scale according to normal or abnormal serum elements levels.

## 3. Results

The concentrations of Zn, Cu, Cu/Zn ratio, Se, Mn, and the total antioxidant status in the serum of the examined patients and healthy volunteers are presented in Table 2.

The concentration of Zn in the serum was significantly lower in patients with degenerative spine disease (0.77 ± 0.22 mg/L) compared to healthy subjects (0.83 ± 0.13 mg/L). There was a significant low correlation (*p* < 0.03) between serum Zn concentration and age in the study group. The concentration of Zn in the blood serum decreased (r = −0.28) with age. We observed that people living in rural areas had a significantly higher (*p* < 0.05) concentration of Zn in the serum compared to those living in the city (0.88 ± 0.36 mg/L and 0.75 ± 0.15 mg/L, respectively). 

We found significantly lower (*p* < 0.05) Cu concentration in the serum of patients with osteoarthritis of the spine (1.1 ± 0.35 mg/L) than in healthy subjects (1.25 ± 0.41 mg/L). Women from the study group had a significantly higher concentration of Cu (1.21 ± 0.37 mg/L) compared to men (0.94 ± 0.26 mg/L). There was also significantly lower Cu concentration in smokers from the study group (1.07 ± 0.35 mg/L) compared to non-smokers from the same group (1.17 ± 0.34 mg/L). 

The median of the Cu/Zn ratio in the study group did not differ significantly from the median of the healthy individuals. There were statistically significant differences between women and men with osteoarthritis of the spine: women had a higher median Cu/Zn ratio than men (1.63 vs. 1.26). However, no significant differences were found between smokers and non-smokers in either the patient group or healthy subjects. There was a significant low positive correlation (r = 0.3, *p* < 0.02) between the Cu/Zn ratio and age in the group of people with osteoarthritis of the spine. Additionally, it was observed that people living in urban areas had a significantly higher (*p* < 0.05) median Cu/Zn ratio compared to those living in the countryside (1.64 vs. 1.31). 

The concentration of Mn and Se in the serum of patients with osteoarthritis of the spine did not differ significantly from healthy people. There were also no significant differences in the concentration of these elements depending on gender and smoking. A significant but negligible correlation (*p* < 0.05) was found between the serum Mn concentration and age in the study group. It was observed that with age, the concentration of Mn in the blood serum decreased (r = −0.23). In the control group, it was noted that the concentration of Mn depends on the concentration of Cu. A positive moderate correlation was found between the concentration of both these elements (r = 0.41; *p* < 0.05).

Serum TAS concentration did not differ significantly between examined and control group. On the other hand, a statistically significant difference was noted between women and men, both in the study group and in healthy subjects. Women from the study and control groups (0.92 ± 0.35 mmol/L and 1.1 ± 0.76 mmol/L) had significantly lower TAS levels compared to men (1.18 ± 0.43 mmol/L and 1.45 ± 0.93 mmol/L, respectively). However, there were no statistically significant differences between smokers and non-smokers in both groups. There was a significant negligible correlation (*p* < 0.05) between TAS concentration and age in the group of people with degenerative spine disease. We noted that with age, the concentration of TAS in the blood serum decreased (r = −0.25). In turn, in healthy people, we noted a significant low negative correlation (r = −0.38, *p* < 0.05) between the concentration of TAS and the concentration of Cu in the serum.

In our study, we did not observe any significant differences in VAS, fat/water ratio in L1 and L5 vertebral body, or in L4/L5 intervertebral disc based on the place of residence or the type of work performed. However, it should be noted that 54% of patients performed sedentary work, of whom, approximately 60% were drivers, dressmakers, and accountants. Among the studied patients, 21% were of normal weight, 54% were overweight, and 24% were obese. There were no significant correlations between VAS or the fat/water ratio in individual spinal structures and the BMI of patients.

The concentration of TAS and selected micronutrients was compared with the ratio of fat to water in individual fragments of the L1 body, the L5 body, and the L4/L5 intervertebral disc obtained in proton magnetic resonance spectroscopy and the VAS scale. The higher the ratio of fat to water, the greater the degree of spine degeneration. The higher the VAS value, the stronger the pain perception of people with osteoarthritis of the spine. 

**Figure 1:**
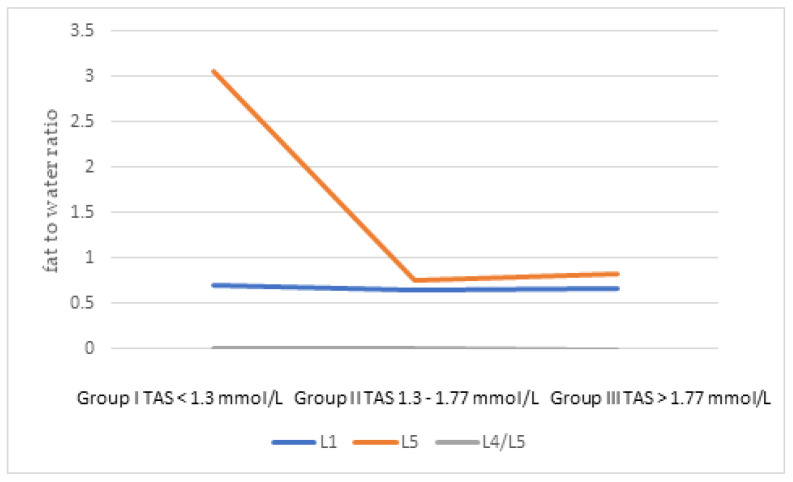
The ratio of fat to water in individual sections of the spine depending on the concentration of Total Antioxidant Status (TAS) in the blood serum.

**Figure 2:**
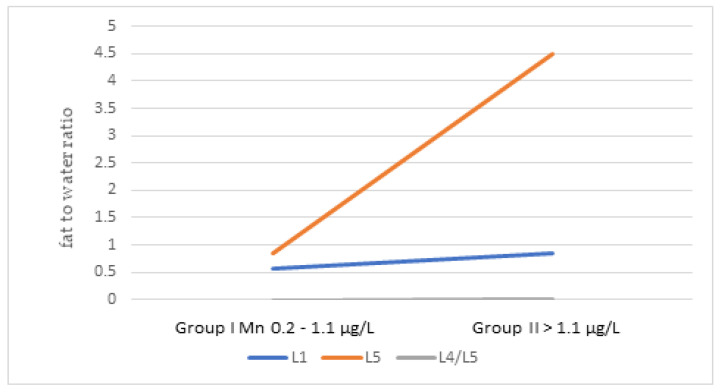
The ratio of fat to water in individual sections of the spine depending on the concentration of Mn in the blood serum.

The ratio of fat to water in individual sections of the spine of patients did not differ significantly depending on the concentration of examined mineral components and TAS in the blood serum, but in the group of patients with TAS below the reference values, the value of the fat/water ratio in the L5 body was significantly higher (Figure 1). In people with elevated serum Mn levels, the fat/water ratio was significantly higher in the L4/L5 segment, which was related to its greater degeneration (Figure 2).

**Figure 3:**
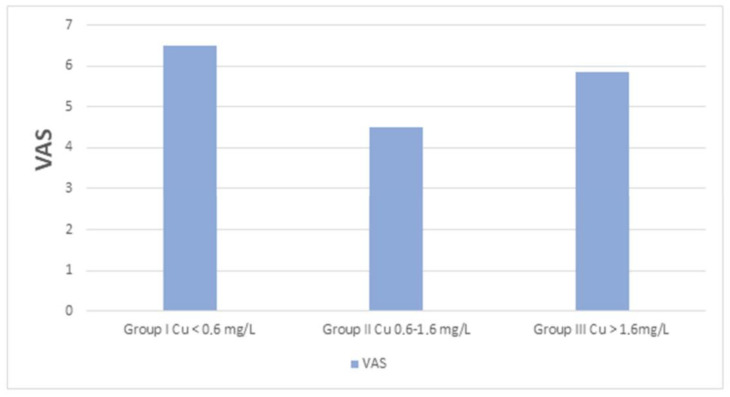
The Visual Analogue Scale (VAS) juxtaposed with the division of people with serum Cu concentration below and within the normal range. Significant differences (*p* < 0.05) in group II vs. group I and III.

The concentration of Zn, Cu, Se, and Mn, as well as TAS, did not significantly affect the perception of pain assessed by VAS. However, patients with normal serum Cu levels experienced less pain (*p* < 0.05) compared to patients with excessively low or high levels of Cu (Figure 3). 

## 4. Discussion

Antioxidant elements, by modulating the inflammatory process, may play an important role in the case of degenerative changes in the spine [11]. 

Zn plays an important role in the development and mineralization of the skeletal system, in bone growth and maturation, and in the inhibition of osteoclast activity and the stimulation of osteoblasts [20,21]. Our research showed that people with degenerative spine disease had a significantly lower concentration of Zn in the blood serum compared to the control group (0.77 ± 0.22 mg/L vs. 0.83 ± 0.13 mg/L). Mahmood obtained similar results and showed that patients with osteoarthritis of the knee had a much lower concentration of Zn in the blood serum compared to healthy people (0.73 ± 0.17 mg/L vs. 2.1 ± 0.54 mg/L) [22]. Convergent results were also obtained by Grennan et al., who observed a significantly lower concentration of Zn in the blood serum of people with osteoarthritis compared to the control group [23]. In our own studies, no significant differences in Zn concentration were found during the division of the studied groups by gender or smoking status. Yazar et al. also did not show significant changes in serum Zn in smokers and non-smokers [24]. We observed inverse association between age and the concentration of Zn in the blood serum. A study by Madej et al. confirmed our results. It proved that in a group of 102 older Europeans, as many as 44% had a deficiency and 20% had a high deficiency of Zn [25]. In addition, in our research, we observed that the place of residence had a significant impact on the concentration of Zn in the serum, as patients living in urban areas had a significantly lower concentration of Zn. This may be associated with different dietary Zn intake, as well as increased use of Zn in the body under oxidative stress caused by higher environmental pollution in urban areas [26]. 

In our study, factors such as place of residence and type of work performed did not affect VAS or fat/water ratio in selected spinal structures. However, we can note that among our patients, the people most-diagnosed with osteoarthritis of the spine were people doing sedentary work, most often drivers, dressmakers, and accountants. Epidemiological studies including 3859 adults showed that sedentary workers constituted the largest group of people with degenerative spine disease [27]. Although we did not show a direct relationship between BMI, and VAS and the degree of spinal degeneration, it should be noted that 78% of patients were overweight or obese. Excessive body weight is one of the risk factors for osteoarthritis of the spine [6,28].

Cu is an essential component that determines proper development and human health. It is a component of many enzymes, including superoxide dismutase or lysyl oxidase, which is the enzyme responsible for the cross-linking of collagen fibers and the formation of cross-links. Disruption of these processes leads to weakening of the bones. Cu increases bone strength and helps to maintain their optimal condition; moreover, its deficiency leads, among other conditions, to bone malformations and increases the risk of osteoporosis in the elderly [21]. Our research showed that people with degenerative spine disease had a significantly lower median concentration of Cu in the serum (0.99 mg/L) compared to healthy people (1.16 mg/L). On the other hand, Mahmood obtained different results. He showed that the concentration of Cu in the blood serum of people with osteoarthritis of the knee was significantly higher compared to the level of Cu in healthy people, which was positively correlated with the duration and severity of the disease [22]. Conforti et al. reported significantly higher levels of Cu in the blood serum of people with rheumatoid arthritis, but not in people with osteoarthritis [29]. In our study, we observed that both in subjects with serum Cu concentration below normal and above the reference range, the degree of pain perception as assessed on the VAS scale was significantly higher. The role of Cu in degenerative diseases of the spine may result from its participation in the construction of connective tissue, bone cover and nervous tissue. Studies have shown the effect of Cu in cell migration, cell adhesion, osteogenesis, chondrogenesis, and angiogenesis in cartilage. It is also involved in many redox metalloenzymes that act on free radicals and influence inflammation. Low levels of Cu in the blood serum may be one of the factors contributing to osteoarthritis of the spine [30,31]. Low Cu concentrations are also shown in diseases such as osteopenia and osteoporosis [32]. Our research showed significant differences in the concentration of Cu in women with degenerative spine disease and in men. The median concentration of Cu in women (1.13 mg/L) was significantly higher than in men (0.88 mg/L). Higher levels of Cu in women as compared to men may be due to the effect of estrogens and increased expression of ATP7A mRNA in the intestine, which lead to increased absorption of this element [33].

There was also a significantly lower Cu concentration in smokers from the test group (0.97 mg/L) compared to non-smokers from the same group (1.11 mg/L). Smoking is usually associated with high levels of cadmium (Cd) in the blood. Exposure to Cd has been associated with aberrant Zn and Cu homeostasis through impairs renal reabsorption of metals [34].

Cu and Zn are two elements that are in competition with each other [35]. Cu/Zn ratio is considered an indicator of oxidative stress and inflammation assessment [36,37]. In our research, the median of the Cu/Zn ratio in the study group did not differ significantly from the median of healthy individuals. On the other hand, there were statistically significant differences between women and men with osteoarthritis of the spine: women had a higher median Cu/Zn ratio than men, which is probably related to the observed higher Cu concentrations in the serum of women. According to data in the literature, the Cu/Zn ratio is higher in women than in men [38,39]. The difference between the sexes in the Cu/Zn ratio can be explained in part by different dietary intake or efficiency of absorption [39] and an increased concentration of Cu and ceruloplasmin in the serum of women, associated with estrogenic effect in women [33]. Additionally, we observed that the place of residence had an impact on the Cu/Zn ratio: it was higher in people living in the city, which may also confirm the influence of oxidative stress and greater environmental pollution [26]. However, no significant differences were found between smokers and non-smokers, both in the group of sick and healthy people. There was a significant association between the Cu/Zn ratio and age in the group of people with degenerative spine disease, which may be associated with higher levels of oxidative stress with age. It was observed that with age, the Cu/Zn ratio in blood serum increased (r = 0.3). Giacconi et al. showed that the variability of the Cu/Zn ratio and the concentration of Zn is influenced to a greater extent by the aging process than the consumption of Zn with diet [37].

Se in the form of selenocysteine, as a component of enzyme proteins, is part of many biochemical pathways. The effect of Se on the skeletal system is mainly related to its antioxidant effect. Se, due to its antioxidant effect, may also be of great importance in osteoarthritis [16]. However, in our study, no statistically significant differences were found in the concentration of Se in the blood serum in the examined and control groups, between men and women, or between smokers and non-smokers in both groups. Mahmood showed that patients with osteoarthritis of the knee had a significantly lower concentration of Se in the blood serum compared to healthy people. It was also correlated with the duration and severity of the disease [22]. The concentration of Se in plasma and serum is influenced by latitude. In China, where Kashin–Beck disease, an inflammatory osteoarthritis, occurs, serum Se concentrations as low as 12–20 µg/L have been reported [40], while in our regions, the levels of Se in the environment, and hence in food and organisms, are higher.

Mn plays an important role in bone formation, fat and carbohydrate metabolism, blood sugar regulation, and calcium absorption. It is the main component and activator of enzymes, including pyruvate carboxylase involved in the antioxidant protection of the body. Due to the role of Mn in the human body, more and more attention is being paid to its importance in osteoarthritis [17]. In our research, no statistically significant differences were found in the concentration of Mn between the study group and the control group, between men and women, or between smokers and non-smokers in both groups. On the other hand, Mahmood showed that the serum Mn concentration was significantly lower in patients with osteoarthritis compared to the concentration of this micronutrient in the control group. These results were negatively and significantly correlated with the duration and severity of the disease [22]. Other studies have shown that diseases such as osteoporosis and secondary osteoarthritis have been observed in people with low blood Mn levels [41]. In our research, it was observed that with the increase in Mn concentration in the blood serum, the fat/water ratio in the L4/L5 intervertebral disc is significantly higher, which is associated with a greater degree of degeneration. This may be influenced by a number of mechanisms. It is known that Mn deficiency in the body can lead to bone malformations [42]. The intervertebral disc consists of type I and II collagen, while the nucleus pulposus consists mainly of type II collagen. In the course of degenerative processes, water content and collagen displacement change, which is visible on MRI and MRS images [43]. Mn is a cofactor of enzymes called glycosyltransferases, which are necessary for the synthesis of proteoglycans filling the space between type II collagen fibers [44]. Mn is also part of the antioxidant enzyme superoxide dismutase (SOD), one of the most important antioxidants [45]. On the other hand, the excess of Mn causes toxic effects, including disturbances in the functioning of the nervous system [46]. In our study, a significant negligible association was found between the Mn concentration and the age of people with degenerative spine disease. It has been shown that Mn levels decrease with age. Similar results were obtained by Oulhote et al., who proved that with age, the concentration of Mn in the blood decreases [47].

Total antioxidant status (TAS) measures the body’s ability to defend itself against free radicals [48]. TAS concentration in people with degenerative spine disease did not differ significantly from healthy people. Our own research investigated the relationship between TAS and age, sex, smoking, VAS, and fat/water ratio in selected structures of the spine. We showed that with age, the concentration of TAS decreases in people with degenerative spine disease. Many authors obtained similar conclusions, including Andriollo-Sanchez et al. [49]. Moreover, both women with osteoarthritis of the spine and healthy women had significantly lower TAS than men from both groups. Similar results were obtained by Hübner-Woźniak et al. They showed that both young and older women had a lower concentration of TAS in the serum compared to young and older men [50]. We observed that patients with TAS concentrations below the reference values had a significantly higher fat/water ratio in L5 vertebral body, which may confirm the important role of oxidative stress in the pathology of degenerative spine disease [9].

Our research has shown that people with osteoarthritis of the spine have a reduced concentration of Zn and Cu in the serum. Moreover, with abnormal Cu concentrations, more pain may occur. On the other hand, there may be an association between elevated Mn levels and decreased serum TAS and a greater degree of degeneration. Therefore, monitoring of the above parameters may be useful in the clinical diagnosis of degenerative disease of the spine. In the case of identified abnormalities, dietary modification or appropriate supplementation can be introduced, which may contribute to the improvement of patients’ health.

Our study has several limitations that should be mentioned. The information on the diet or food frequency questionnaires (FFQ) both in the study and control group were unavailable. Eating habits could have impacted the obtained results. We have no information about the degree of education, medications used, or the level of physical activity beyond the type of work performed; the relatively small group of subjects made it impossible to consider the above factors in our research. Unfortunately, we also did not have demographic data such as type of job or education from the subjects from the control group. We also did not have imaging findings of the spine from healthy people. The research could be extended by a comparison of the obtained results of laboratory tests with the intensity of degenerative changes in the spine assessed in the Modic scale; unfortunately, we do not have complete data.

## 5. Conclusions

Reduced levels of antioxidants through the development of oxidative stress can contribute to the development of osteoarthritis of the spine. The research presented in this paper highlights the importance of examined elements in patients with degeneration of the spine. This research may be important in establishing the right diet for people with osteoarthritis of the spine, as well as in supporting new methods of treating this disease based on antioxidant ingredients. The conducted studies also indicate the usefulness of MRS in the diagnosis of this disease, as this short and non-invasive complementary MRI examination provides much additional information about the severity of lesions.

## Figures and Tables

**Table 1:** Characteristics of the study groups.

	Examined Group *n* = 90		Control Group *n* = 40	
Age (years) mean ± SD (range)	48.2 ± 12.7 (21–73)		45.8 ± 16.0 (21–83)	
Gender	Female 58%	Male 42%	Female 70%	Male 30%
BMI (kg/m^2^) mean ± SD (range)	28.1 ± 4.1 (20.1–38.3)		26.2 ± 3.8 (20.7–35.2)	
Smoking cigarettes	Yes 31%	No 69%	Yes 25%	No 75%

*n*—number of participants, SD—standard deviation; BMI—body mass index.

**Table 2:** The concentrations of zinc, copper, selenium, and manganese, Cu/Zn ratio, and total antioxidant status of patients with degenerative spine disease and control group.

	Examined Group Mean ± SD Min–Max Median (Q1–Q3)		Control Group Mean ± SD Min–Max Median (Q1–Q3)		*p* Value
	Total		Total		
Zinc (mg/L)	0.77 ± 0.22 (0.41–2.1) 0.76 (0.64–0.85)		0.83 ± 0.13 (0.55–1.09) 0.81 (0.74–0.91)		0.001 *
	F (a)	M (b)	F (c)	M (d)	
	0.74 ± 0.15	0.80 ± 0.28	0.83 ± 0.13	0.85 ± 0.14	ns
	(0.41–1.08) 0.76 (0.64–0.83)	(0.50–2.10) 0.75 (0.63–0.86)	(0.55–1.09) 0.81 (0.74–0.99)	(0.67–1.07) 0.81 (0.74–0.91)	
	Smokers (a)	Non-smokers (b)	Smokers (c)	Non-smokers (d)	
	0.81 ± 0.39 (0.41–2.1) 0.74 (0.67–0.88)	0.75 ± 0.12 (0.53–0.98) 0.77 (0.66–0.83)	0.87 ± 0.22 (0.55–1.09) 0.92 (0.67–1.07)	0.79 ± 0.09 (0.56–0.99) 0.79 (0.75–0.86)	ns
Copper (mg/L)	1.1 ± 0.35 (0.53–2.3) (0.99) (0.88–1.2)		1.25 ± 0.41 (0.81–2.36) 1.16 (0.92–1.49)		0.04 *
	F (a)	M (b)	F (c)	M (d)	
	1.21 ± 0.37 (0.75–2.3) 1.13 (0.96–1.33)	0.94 ± 0.26 (0.53–2) 0.88 (0.81–0.99	1.22± 0.39 (0.76–2.3) 1.1 (0.9–1.49)	1.2 ± 0.48 (0.7–2.36) 1.1 (0.86–1.4)	a vs. b < 0.00001 *
	Smokers (a)	Non-smokers (b)	Smokers (c)	Non-smokers (d)	
	1.07 ± 0,35 (0.67–2.03) 0.97 (0.88–1.15)	1.17 ± 0.34 (0.76–2.22) 1.11 (0.9–1.2)	(1.2 ± 0.39) (0.86–1.78) 1.15 (0.86–1.49)	1.16 ± 0.39 (0.7–2.31) 1.07 (0.86–1.29)	a vs. b < 0.05 *
Cu/Zn ratio	1.54± 0.5 (0.35–2.98) 1.48 (1.15–1.81)		1.56± 0.65 (0.80–3.44) 1.4 (1.12–1.84)		ns
	F (a)	M (b)	F (c)	M (d)	
	1.73 ± 0.5 (0.85–2.98) 1.63 (1.37–2.05)	1.29 ± 0.39 (0.35–2.17) 1.26 (1.05–1.48)	1.57 ± 0.61 (0.82–3.32) 1.44 (1.15–1.87)	1.54 ± 0.76 (0.8–3.49) 1.35 (1.08–1.57)	a vs. b = 0.00006 *
	Smokers (a)	Non-smokers (b)	Smokers (c)	Non-smokers (d)	
	1.49 ± 0.58 (0.35–2.78) 1.4 (1.11–1.72)	1.61 ± 0.45 (0.94–2.98) 1.49 (1.31–1.88)	1.61 ± 0.96 (0.81–1.32) 1.23 (0.98–2.12)	1.52 ± 0.55 (0.8–3.11) 1.4 (1.15–1.81)	ns
Selenium (μg/L)	77.3 ± 21.5 (36.9–189.1) 74.8 (66.3–86.1)		78.3 ± 14.5 (50.9–112.8) 75.8 (69.8–85.2)		ns
	F (a)	M (b)	F (c)	M (d)	
	78.6 ± 22.7 (48–189.1) 74.6 (66.9–86.1)	(75.4 ± 19.8 (36.9–133.7) 77.6 (64.3–86.4)	78.1 ± 14 (59.8–112.8) 75.2 (69.7–84.2)	78.6 ± 16.7 (50.9–106.8) 76.7 (70.5–89.1)	
	Smokers (a)	Non-smokers (b)	Smokers (c)	Non-smokers (d)	
	72.6 ± 16.1 (36.9–103.7) 74.1 (60.4–84)	73.6 ± 15.1 (42.6–104.6) 73.6 (66.5–82.4)	76.8 ± 9.1 (70.5–94.9) 73.9 (71.2–76.1)	76.7 ± 12.9 (59.8–112.6) 76 (68.2–83.6)	
Manganese (mg/L)	1.25 ± 1.28 (0.23–9.76) 1.06 (0.79–1.32)		1.7 ± 1.98 (0.27–7.91) 0.81 (0.67–1.72)		ns
	F (a)	M (b)	F (c)	M (d)	
	1.19 ± 1.13 (0.22–8.24) 1.06 (0.76–1.28)	1.34 ± 1.48 (0.36–9.76) 1.04 (0.82–1.36)	1.35 ± 1.39 (0.27–5.4) 0.77 (0.64–1.03)	2.43 ± 2.82 (0.58–7.9) 0.94 (0.75–2.1)	
	Smokers (a)	Non-smokers (b)	Smokers (c)	Non-smokers (d)	
	1.43 ± 1.44 (0.51–8.24) 1.21 (0.92–1.38)	1.4 ± 1.51 (0.53–9.76) 1.1 (0.88–1.34)	1.99 ± 2.64 (0.59–6.71) 0.94 (0.74–1.03)	1.97 ± 2.21 (0.49–7.91) 0.81 (0.66–2.91)	
Total antioxidant status (mmol/L)	1.03 ± 0,41 (0.1–2.14) 1.01 (0.71–1.28)		1.39 ± 0.84 (0.71–3.5) 0.97 (0.88–1.58)		ns
	F (a)	M (b)	F (c)	M (d)	
	0.92± 0.35 (0.1–1.74) 0.97 (0.59–1.17)	1.18 ± 0.43 (0.62–2.14) 1.12 (0.75–1.4)	1.1 ± 0.76 (0.51–3.5) 0.82 (0.63–1.04)	1.45 ± 0.93 (0.87–3.46 1.06 (0.93–1.636)	a vs. b < 0.01 * c vs. d < 0.05 *
	Smokers (a)	Non-smokers (b)	Smokers (c)	Non-smokers (d)	
	0.99 ± 0.38 (0.31–1.78) 0.98 (0.71–1.29)	1.15 ± 0.41 (0.49–2.14) 1.157 (0.86–1.31)	0.79 ± 0.14 (0.6–0.93) 0.82 (0.7–0.9)	1.35 ± 1 (0.51–3.5) 0.3 (0.63–1.85)	ns

SD—standard deviation, F—female, M—male, Q1—lower quartile, Q3—upper quartile, *p*—significance level, *—statistically significant differences, ns—no significance.

## Data Availability

The data presented in this study are available on request from the corresponding author. The data are not publicly available due to privacy of ethical.
